# The freshwater crabs of Danum Valley Conservation Area in Sabah, East Malaysia, with a description of a new species of *Thelphusula* Bott, 1969 (Crustacea, Brachyura, Gecarcinucidae, Potamidae, Sesarmidae)

**DOI:** 10.3897/zookeys.760.24787

**Published:** 2018-05-28

**Authors:** Peter K. L. Ng, Paul Y. C. Ng

**Affiliations:** 1 Lee Kong Chian Natural History Museum, National University of Singapore, 2 Conservatory Drive, Singapore 117377, Republic of Singapore; 2 205 River Valley Road, #16-53, Singapore 238274, Republic of Singapore

**Keywords:** Taxonomy, Borneo, *Thelphusula capillodigitus*, *Arachnothelphusa*, *Terrathelphusa*, *Parathelphusa*, *Isolapotamon*, *Geosesarma*, phytotelm

## Abstract

Seven species of freshwater crabs from three families are recorded from and around the Danum Valley Conservation Area in Sabah, Malaysian Borneo: *Thelphusula capillodigitus* **sp. n.**, *Thelphusula dicerophilus* Ng & Stuebing, 1990, *Arachnothelphusa terrapes* Ng, 1991, *Terrathelphusa secula* Ng & Tan, 2015, *Parathelphusa valida* Ng & Goh, 1987 (new record) (Gecarcinucidae); *Isolapotamon ingeri* Ng & Tan, 1998 (Potamidae); and *Geosesarma danumense* Ng, 2002 (Sesarmidae). The new species of *Thelphusula* Bott, 1979, can be distinguished from all congeners by a unique combination of morphological features, most notably the presence of dense patches of short setae on the fingers of the adult male chelipeds, as well as the structure of the male first gonopod. *Arachnothelphusa terrapes* is confirmed to be a phytotelm species. A key to all species in the conservation area is provided.

## Introduction

Danum Valley Conservation Area, northeastern Borneo, in the Malaysian state of Sabah contains over 400 square kilometres of pristine rainforest and is a key conservation area on the island (Hazebroek et al. 2011). The first author has been involved in studies of the freshwater decapod crustacean fauna in the area since the late 1980s, and five species of freshwater and semiterrestrial crabs have been reported thus far: *Thelphusula dicerophilus* Ng & Stuebing, 1990, *Arachnothelphusa terrapes* Ng, 1991, *Terrathelphusa secula* Ng & Tan, 2015 (Gecarcinucidae Rathbun, 1904); *Isolapotamon ingeri* Ng & Tan, 1998 (Potamidae Ortmann, 1896); and *Geosesarma danumense* Ng, 2002 (Sesarmidae Dana, 1851) (Ng 1991, 2002; Ng and Stuebing 1990; Ng and Tan 1998, 2015).

Here we review and add to the freshwater crab fauna of the Danum Valley Conservation Area. Specimens of a recently collected *Thelphusula* Bott, 1969, from Danum Valley proved to be a new species. While superficially resembling *T. hulu* Tan & Ng, 1997, from the Maliau Basin in Sabah in morphology and habits, it can easily be distinguished from this and all congeners by its setose male cheliped dactyli, as well as a number of other carapace features. It is here described as *T. capillodigitus* sp. n. In addition, *Parathelphusa valida* Ng & Goh, 1987, is added to the fauna for the area. Observations on the ecology of *Arachnothelphusa terrapes* Ng, 1991, originally described from Danum Valley, are also provided, showing that it is only the second confirmed tree-hole crab in South East Asia. A key to the seven species of Gecarcinucidae, Potamidae, and Sesarmidae in the Danum Valley Conservation Area is provided.

## Materials and methods

The terminology used follows that in Ng (1988) and Davie et al. (2015). Measurements provided, in millimetres, are of the maximum carapace width and length, respectively. The abbreviations G1 and G2 are used for the first and second male gonopods, respectively. Specimens examined are deposited in the Zoological Reference Collection (ZRC) of the Lee Kong Chian Natural History Museum, National University of Singapore.

## Systematic account

### Family Gecarcinucidae Rathbun, 1904

#### Genus *Thelphusula* Bott, 1969

##### Type species.

Potamon (Geothelphusa) buergeri De Man, 1899, by original designation.

##### Remarks.

*Thelphusula* Bott, 1969, was established for Potamon (Geothelphusa) buergeri De Man, 1899, and currently contains 11 species (Ng and Tan 1998; Ng et al. 2008; Grinang and Ng 2014), all from Borneo. Of these, five species are found in Sabah: *T. dicerophilus* Ng & Stuebing, 1990, *T. hulu* Tan & Ng, 1997, *T. luidana* (Chace, 1938), *T. sabana* Tan & Ng, 1998, and *T. tawauensis* Tan & Ng, 1998. The genus is characterized by its quadrate carapace shape, the epibranchial tooth being poorly developed or absent, the posterolateral margins which are subparallel, the ambulatory legs being not prominently elongate, the G1 being slender with a relatively long and cylindrical terminal segment, and a relatively short G2 which has a short flagellum (Tan and Ng 1998).

##### Thelphusula capillodigitus sp. n.

Taxon classificationAnimaliaDecapodaGecarcinucidae

http://zoobank.org/EBC4CF72-6C54-408A-ADD1-60190EF3FDAF

**Figure 1.**
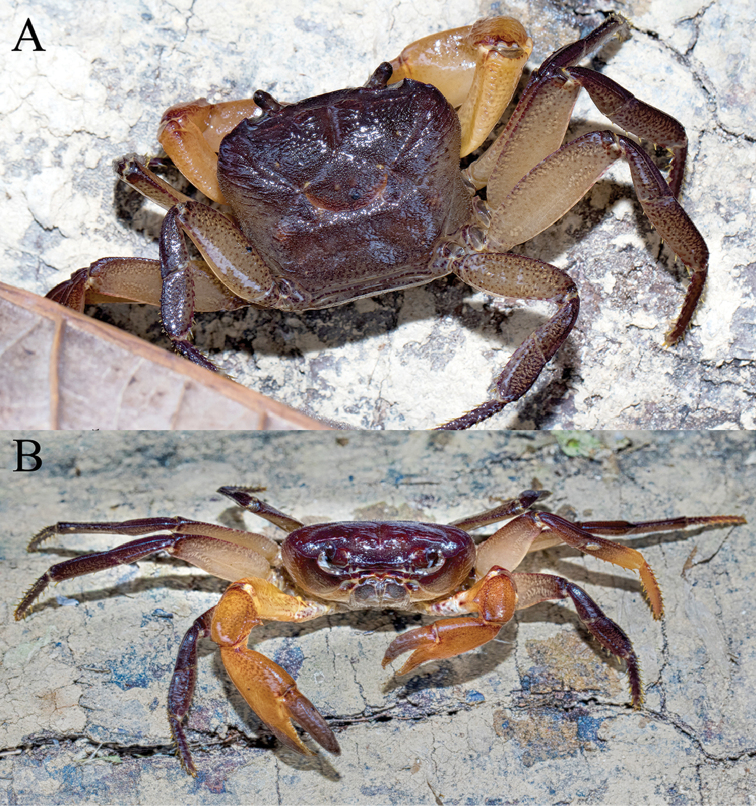
*Thelphusula capillodigitus* sp. n., colour in life, holotype male (23.9 × 18.4 mm) (ZRC 2017.1294), Sabah. **A** dorsal view **B** frontal view (photographs Dennis Sim).

**Figure 2.**
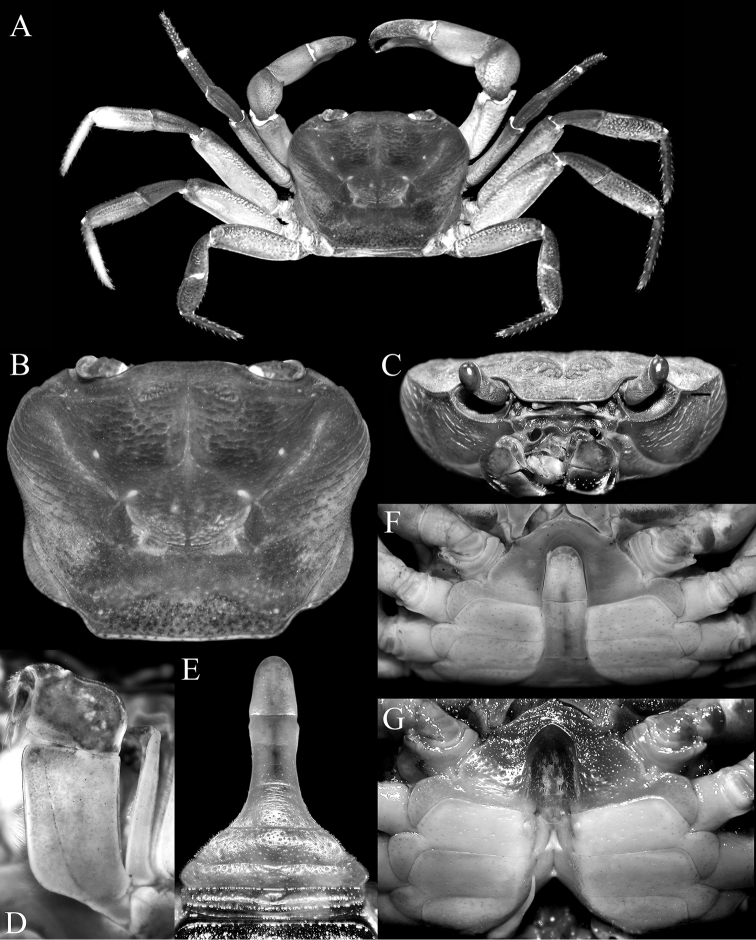
*Thelphusula capillodigitus* sp. n., holotype male (23.9 × 18.4 mm) (ZRC 2017.1294), Sabah. **A** overall view **B** dorsal view of carapace **C** frontal view of cephalothorax **D** left third maxilliped **E** pleon **F** anterior thoracic sternum and pleon **G** sternopleonal cavity.

**Figure 3.**
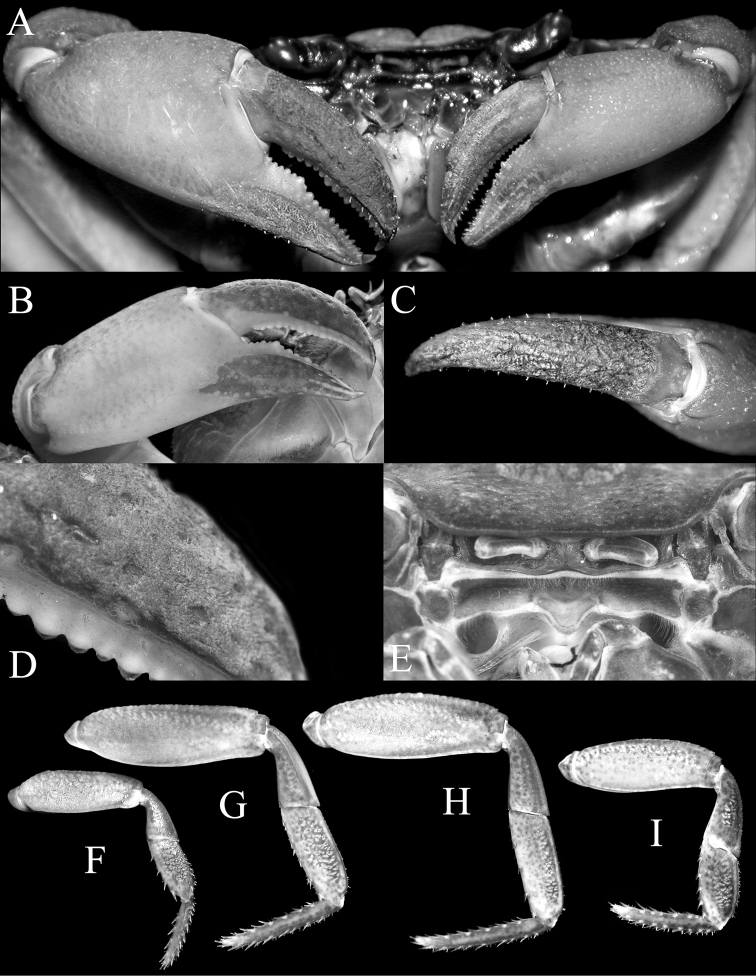
*Thelphusula capillodigitus* sp. n., holotype male (23.9 × 18.4 mm) (ZRC 2017.1294), Sabah. **A** outer views of chelae **B** outer view of right chela **C** dorsal view of dactylus of right chela **D** surface of dactylus showing dense short setae **E** epistome **F–I** first to fourth ambulatory legs, respectively (all to same scale).

**Figure 4.**
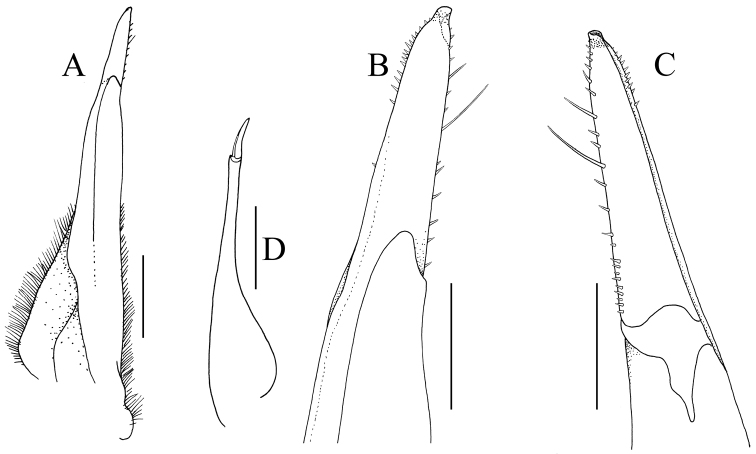
*Thelphusula capillodigitus* sp. n., holotype male (23.9 × 18.4 mm) (ZRC 2017.1294), Sabah. **A** left G1 (ventral view) **B** distal part of left G1 (ventral view) **C** distal part of left G1 (doral view) **D** left G2 (ventral view). Scale bars: 1.0 mm (**A, D**); 0.5 mm (**B, C**).

Figures 1 , 2 , 3 , 4

###### Material examined.

Holotype: male (23.9 × 18.4 mm) (ZRC 2017.1294), coll. Danum Valley, Lahad Datu, Sabah, Borneo, Malaysia, 22 July 2017. Paratypes: 1 male (18.8 × 15.5 mm) (ZRC 2017.1295), same data as holotype; 1 male (19.9 × 16.4 mm) (ZRC 2009.0080), in pitfall trap, Danum Valley Research Centre, Sabah, coll. C. Colón, October 1996. Others: 3 juveniles (3.7 × 3.0 mm, 6.5 × 5.4 mm, 6.7 × 5.5 mm), 1 young female (10.7 × 9.1 mm) (ZRC 1990.0548–0551), Danum Valley Research Centre, Lahad Datu, Sabah, coll. R. Stuebing, 23 July 1989.

###### Diagnosis.

Carapace broader than long, not raised; dorsal surface with regions clearly demarcated; frontal median triangle absent (Figs 2A–C, 3E); epibranchial tooth low, distinct, separated from external orbital tooth by shallow cleft; epigastric regions raised, rugose, not cristate; postorbital cristae low, distinct, rugose, not confluent with epigastric cristae, not reaching anterolateral margin; cervical grooves and H-shaped gastric depression deep; gastric regions with prominent transverse striae; antero- and posterolateral regions with strong oblique striae (Fig. 2A, B); median lobe on posterior margin of epistome triangular, tip rounded (Figs 2C, 3E). Third maxilliped with subrectangular ischium, distinctly longer than broad (Fig. 2D). Chelipeds with outer surface of palm almost smooth, dorsal and lateral surfaces of adult male dactylus covered with dense short setae (Fig. 3A–D). Ambulatory legs not prominently elongate, dorsal margin of merus gently serrated (Figs 2A, 3F–I). Thoracic sternum with surface evenly pitted to smooth, sternopleonal cavity reaching imaginary line joining anterior edges cheliped coxae (Fig. 2F, G); pleon distinctly T-shaped, somite 6 rectangular, slightly more than twice as long as broad, telson triangular, longer than broad (Fig. 2E, F). G1 relatively slender, almost straight; terminal segment approx. a quarter length of subterminal segment (Fig. 4A–C). G2 approx. two-thirds length of G1, distal article short (Fig. 4D).

###### Description of male holotype.

Carapace broader than long, not raised; dorsal surface gently convex, regions clearly demarcated, covered with very short setae which does not obscure surface; frontal margin almost straight, without distinct median concavity, not deflexed, approx. a third carapace width; frontal median triangle absent (Figs 2A–C, 3E); anterolateral margin not clearly separated from posterolateral margin; external orbital tooth low, broadly triangular; epibranchial tooth low but distinct, separated from external orbital tooth by shallow cleft; postfrontal surface slightly rugose; postorbital region surface; epigastric regions raised, rugose, not cristate, divided into 2 parts by narrow, deep median groove; postorbital cristae low but distinct, sharp, not confluent with epigastric cristae, not reaching anterolateral margin; cervical grooves and H-shaped gastric depression deep; gastric regions with prominent transverse striae; antero- and posterolateral regions with strong oblique striae; posterolateral margins concave, gently converging towards posterior carapace margin; posterior carapace margin straight (Fig. 2A, B); pterygostomial, suborbital, sub-branchial, and subhepatic regions covered with striae (Fig. 2C); orbits large, eyes occupying entire width, supra and suborbital margins entire, cristate; eyes pigmented, well developed, peduncle with low, sinuous median ridge (Fig. 2B, C); median lobe on posterior margin of epistome triangular, tip rounded, lateral margins sinuous (Figs 2C, 3C).

Mandibular palp 2-segmented, terminal one distinctly bilobed. Third maxilliped covering majority of buccal cavity when closed; ischium subrectangular, distinctly longer than broad, with shallow submedian groove; merus quadrate, slightly broader than long; exopod long, slender, reaching median part of merus, flagellum long, exceeding width of merus (Fig. 2D).

Chelipeds asymmetrical, right larger; surface of merus slightly rugose, relatively long, trigonal in cross section, margins without teeth or spines; carpus surface distinctly rugose, subovate, inner distal angle with sharp spine with basal tubercle; palm relatively stout, longer than broad, outer surface slightly rugose to almost smooth; fingers subequal in length to palm, dactylus marginally longer than pollex, curving inwards, cutting margin of fingers lined with numerous denticles, fingers pitted (Figs 2A, 3A–D); dorsal and lateral surfaces of most of dactylus covered with dense short setae; lateral surface of pollex with mat of short, relatively less dense setae; tips of fingers strongly curved, corneous, glabrous (Fig. 3B–D).

Ambulatory legs not prominently elongate, third pair longest, fourth leg shortest; segments laterally flattened laterally, surfaces mildly rugose; dorsal margin of merus gently serrated, no visible subdistal tooth; carpus of first to third legs with low median ridge, absent on carpus of fourth leg; margins of propodus and dactylus lined with numerous short spines (Figs 2A, 3F–I).

Thoracic sternum surface evenly pitted to smooth; sternites 1 and 2 completely fused forming triangular structure; suture separating sternites 2 and 3 relatively shallow, sinuous, medially convex with lateral parts concave (towards buccal cavity); sternites 3 and 4 completely fused; sternopleonal cavity almost reaching imaginary line joining anterior edges cheliped coxae, near suture between sternites 2 and 3; part of sternite 8 exposed when pleon closed; tubercle of male pleonal locking mechanism prominent, peg-like, on anterior third of sternite 5 (Fig. 2F, G).

Pleon distinctly T-shaped; somite 1 short, broad, reaching coxae of fourth ambulatory legs; somite 2 slightly longer than somite 1, as broad as somite 1; somite 3 short, broadest, with prominently convex lateral margins; somites 4 and 5 trapezoidal; somite 5 notably narrower than 4, trapezoidal with concave lateral margins; somite 6 rectangular, slightly more than twice as long as broad, lateral margins concave; telson triangular, longer than broad, tip rounded (Fig. 2E, F).

G1 relatively slender, entire structure almost straight; terminal and subterminal segments clearly separated; terminal segment relatively short, approx. a quarter length of subterminal segment, cylindrical with tip tapering to subtruncate tip, margins with short stiff setae, surface just before tip with numerous squamiform setae; lower half of subterminal segment with numerous short setae (Fig. 4A–C). G2 approx. two-thirds length of G1; basal segment long; distal segment short (Fig. 4D).

###### Variation.

Unlike the male holotype (the largest specimen), the degree and extent of the setation on the fingers of the chelae of the two smaller paratype males are the same in both chelipeds. Male specimens less than 15 mm in carapace width do not have the setae on the fingers of the chelae. The outer surface of the chela in smaller specimens is also relatively more rugose compared to larger ones.

###### Etymology.

The name is derived from the Latin *capillus* for hair and *digitus* for finger. The name is used as a noun in apposition.

###### Colour.

In life, the carapace is mostly dark reddish brown; the sub-branchial regions, third maxillipeds, pleon and thoracic sternum is pale yellow; the ambulatory legs dark brown, faintly marmorated, with exception of pale yellow, faintly spotted merus; and the chelipeds are yellowish orange, with the inner surfaces paler and the setose patches on the surface of the male fingers light brown (Fig. 1).

###### Remarks.

*Thelphusula capillodigitus* sp. n. can easily be distinguished from all congeners by the adult male possessing dense setae on the dorsal surfaces of the fingers of the chelipeds (Fig. 3B–D), a character also absent in genera allied to *Thelphusula*: *Adeleana* Bott, 1969, *Balssiathelphusa* Bott, 1969, *Stygothelphusa* Ng, 1989, *Arachnothelphusa* Ng, 1991, and *Coccusa* Tan & Ng, 1998 (cf. Bott 1969, 1970; Ng 1989b, 1991; Tan and Ng 1998; Ng and Guinot 2014). The absence of a clearly discernible frontal median triangle is a character *T. capillodigitus* shares with *T. pueh*, *T. cristicervix* and *T. styx*, but it can be distinguished from them by the presence of setose patches on the fingers of the adult male chelipeds (Fig. 3B–D) as well as a G1 which is only slightly curved with a relatively shorter terminal segment that is approx. a third the length of the subterminal segment (Fig. 4A–C). In contrast, the G1s in *T. pueh* and *T. cristicervix* possess a prominently curved terminal segment which is proportionately longer, being approx. half the length of the subterminal segment (cf. Ng and Grinang 2014: fig 3). In *T. styx*, the G1 has a relatively broader subterminal segment with the terminal segment distinctly upturned (cf. Ng 1989a: figs 2E, F). *Thelphusula capillodigitus* can further be distinguished from *T. styx* by its relatively more shallow cervical grooves which end at the H-shaped median depression with a level and straight frontal margin (Fig. 2B) (versus with deeper and distinctly longer cervical grooves that extend to the posterolateral region of the carapace, and the frontal margin deflexed in *T. styx*; cf. Ng 1989a: fig.1). The carapace of *T. capillodigitus* is gently convex (Fig. 2B, C) whereas in both *T. pueh* and *T. cristicervix*, the carapaces are distinctly inflated (cf. Ng and Grinang 2014: figs 1C, 2C). In addition, *T. pueh*, *T. cristicervix*, and *T. styx* are only known from Sarawak.

In the general form of the carapace (not raised and relatively low) and relatively shorter ambulatory legs, *T. capillodigitus* most closely resembles *T. sabana* from Lahad Datu and *T. hulu* from the Maliau Basin, both in Sabah. Other than in the setose adult male cheliped fingers, *T. capillodigitus* can also be distinguished by the gastric regions prominently lined with transverse striae (Fig. 2B) (versus gastric regions rugose to smooth in *T. hulu*; cf. Tan and Ng 1997: fig. 5); the absence of a frontal median triangle (Figs 2C, 3E) (versus frontal median triangle distinct in *T. hulu*; Tan and Ng 1997: fig. 3B); the ischium of the third maxilliped being relatively longer (Fig. 2D) (versus ischium relatively shorter in *T. hulu*; cf. Tan and Ng 1997: fig. 3D); the outer surface of chela being almost smooth (Fig. 3A, B) (versus covered with prominent striae and scattered granules in *T. hulu*; cf. Tan and Ng 1997: fig. 3C); the male pleonal somite 6 being proportionately longer (Fig. 2E) (versus male pleonal somite 6 proportionately shorter in *T. hulu*; cf. Tan and Ng 1997: fig. 4B); and the G1 being almost straight (Fig. 4A–C) (versus G1 terminal segment strongly curved outwards in *T. hulu*; cf. Tan and Ng 1997: fig. 4C, D). *Thelphusula capillodigitus* resembles *T. sabana* from Lahad Datu in possessing strong striae and granules on the carapace surface, but can be separated by lacking a frontal median triangle (Figs 2C, 3E) (versus frontal median triangle discernible but incomplete in *T. sabana*; cf. Tan and Ng 1998: fig. 3C); the male pleonal somite 6 is proportionately longer (Fig. 2E) (versus male pleonal somite 6 proportionately shorter in *T. sabana*; cf. Tan and Ng 1998: fig. 3B); and the G1 is almost straight with a short terminal segment (Fig. 4A–C) (versus G1 prominently curved outwards with the terminal segment very long in *T. sabana*; cf. Tan and Ng 1998: fig. 3D–G).

Two other species of *Thelphusula* are present in Sabah, *T. dicerophilus* (which occurs in the same area as *T. capillodigitus*) and *T. tawauensis* which occurs to the east. *Thelphusula capillodigitus* can be separated from *T. dicerophilus* easily by its relatively flatter carapace (Fig. 2B, C) (versus carapace very high and raised in *T. dicerophilus*; Fig. 6B; cf. Ng and Stuebing 1990: pl. 1B); and from *T. tawauensis* by the gastric regions covered with prominent striae and the frontal median triangle being absent (Figs 2C, 3E) (versus gastric regions smooth with the frontal median triangle prominent in *T. tawauensis*; cf. Tan and Ng 1998: fig. 4A, C).

*Thelphusula capillodigitus* was collected in a clear flowing shaded jungle stream with an average temperature range of 26–28 degrees Celsius and near neutral pH. All specimens were collected during the day, under rocks, and appear to be mostly aquatic in habits, although one specimen was collected from a pitfall trap (ZRC 2009.0080). *Parathelphusa valida* was also present in the same stream in larger numbers. The presence of a second species of *Thelphusula* in Danum Valley is not surprising, considering that *T. capillodigitus* has more aquatic habits than *T. dicerophilus* (see next species).

##### Thelphusula dicerophilus

Taxon classificationAnimaliaDecapodaGecarcinucidae

Ng & Stuebing, 1990

Figure 6A, B

Thelphusula dicerophilus Ng & Stuebing, 1990: 46, fig. 1, pl. 1; Tan and Ng 1998: 813, fig. 6C; Ng et al. 2008: 73.

###### Material examined.

Holotype: male (14.0 × 12.0 mm) (ZRC 1989.3588), in pitfall trap, adjacent to pool of rhinoceros mud wallow, Danum Valley, Lahad Datu, Sabah, ca. 4°55'N 117°46', coll. R. Stuebing, 4 March l988. Paratypes: 1 female (18.3 × 15.0 mm), 2 juveniles (ZRC 1989.3592–3594), in pitfall trap, adjacent to pool of rhinoceros mud wallow, Danum Valley, Lahad Datu, Sabah, ca. 4°55'N 117°46', coll. R. Stuebing, 1 March 1988; 1 male (18.5 × 15.4 mm) (ZRC 1989.3591), in mist net in rhinoceros mud wallow pool, Danum Valley, Lahad Datu, Sabah, ca. 4°55'N 117°46', coll. R. Stuebing, 2 March l988; 1 male, 1 female (ZRC 1989.3589–3590), in rhinoceros mud wallow pool, Danum Valley, Lahad Datu, Sabah, ca. 4°55'N 117°46', coll. R. Stuebing, 2 March l988. Others: 1 female (11.0 × 9.5 mm) (ZRC 1997.0138), in pitfall trap, Danum Valley Field Centre, Lahad Datu, Sabah, coll. C. Colón, 14 October 1996; 1 male (16.4 × 13.5 mm) (ZRC 1997.0140), in pitfall trap, Danum Valley Field Centre, Lahad Datu, Sabah, coll. C. Colón, 10 October 1996; 1 male (20.0 × 15.8 mm) (ZRC 1997.0141), in pitfall trap, Danum Valley Field Centre, Lahad Datu, Sabah, coll. C. Colón, 17 October 1996; 1 male (22.0 × 17.6 mm) (ZRC 1997.0142), in pitfall trap, Danum Valley Field Centre, Lahad Datu, Sabah, coll. C. Colón, 19 October 1996; 1 male (10.5 × 9.0 mm), 1 female (17.8 × 14.9 mm) (ZRC 2017.1272), in mud and leaves under wooden walk-way, Orchid Trail, Danum Valley Field Centre, Lahad Datu, Sabah, at night, coll. local rangers, 20 July 2017; 1 female (26.1 × 21.3 mm) (ZRC 2017.1047), in pool along wooden walkway at night, Orchid Trail, Danum Valley Field Centre, Lahad Datu, Sabah, at night, coll. local rangers, 20 July 2017; 1 female (24.0 × 17.5 mm) (ZRC 1997.0139), Kunak, Baturong, Binuang River, Lahad Datu, Sabah, coll. R. Stuebing, 20 March 1989.

###### Colour.

In life, the carapace is reddish brown with the ambulatory legs lighter in colour; the chelipeds are orangish red with the fingers pale-yellow (Fig. 6A, B).

###### Remarks.

The present series of specimens do not change the original description of this species in any way. The species does grow substantially larger than the type series, with the largest specimen here, a female measuring 26.1 × 21.3 mm (ZRC 2017.1047).

The available collection data indicates *T. dicerophilus* is a semiterrestrial nocturnal species and forages on the forest floor, usually in wet, swampy areas, digging burrows in the soft substrate; they were often caught in pitfall traps set near these areas (see also Ng and Stuebing 1990). This contrasts with the more aquatic habits of its congener in Danum Valley, *T. capillodigitus* sp. n.

#### Genus *Arachnothelphusa* Ng, 1991

##### Type species.

Potamon (Potamon) melanippe De Man, 1899, by original designation.

###### Arachnothelphusa terrapes

Taxon classificationAnimaliaDecapodaGecarcinucidae

Ng, 1991

Figure 5

Arachnothelphusa terrapes Ng, 1991: 8, figs 3–6; Ng et al. 2008: 69.

####### Material examined.

Holotype: male (17.6 × 13.3 mm) (ZRC 1992.7918), Danum Valley Field Centre, station 507, in dry stump on ridge, Lahad Datu, Sabah, Borneo, leg. H.K. Voris, 23 October 1990. Paratype: female (25.7 × 18.6 mm) (ZRC 1992.7919), Danum Valley, Lahad Datu, Sabah, Borneo, leg. S.C. Choy, 21 July 1989. Others: 1 male (30.8 × 20.5 mm), 1 female (30.1 × 20.5 mm, with 26 juvenile crabs) (ZRC 2017.1205), from water-filled tree buttress, ca. 35 cm above ground Danum Valley, Lahad Datu, Sabah, Borneo, Malaysia, 20 July 2017.

####### Comparative material.

*Arachnothelphusa kadamaiana* (Borradaile, 1900): 1 female (23.2 × 17.1 mm) (ZRC 2009.0094), Poring, Basin 1A, Sabah, Malaysia, Borneo, coll. R.F. Inger et al., 12 August 1992; 3 males (21.1 × 15.8 mm, 22.8 × 16.5 mm, 25.3 × 18.5 mm) (ZRC 2002.0097), Crocker Range, Sabah, 5°27'N 116°03'E, coll. I. Das, 24 April 2001. Arachnothelphusa aff. kadamaiana: 1 female (19.0 × 14.2 mm) (ZRC 2002.0098), Bako National Park, Sarawak, coll. I. Das and L. Grismer, 27 March 2001. *Arachnothelphusa merarapensis* Grinang, Pui & Ng, 2015: Holotype male (22.5 × 16.8 mm) (ZRC 2016.0297), water-filled tree-hole, ca. 100 cm above ground, steep dipterocarp forest, Merarap Hot Spring Resort, Lawas, northern Sarawak, Malaysia, Borneo, 4°22'25.4"N, 115°26'10.1"E, 485 m asl, coll. J. Grinang and Y.M. Pui, 31 October 2014.

####### Colour.

**Figure 5.**
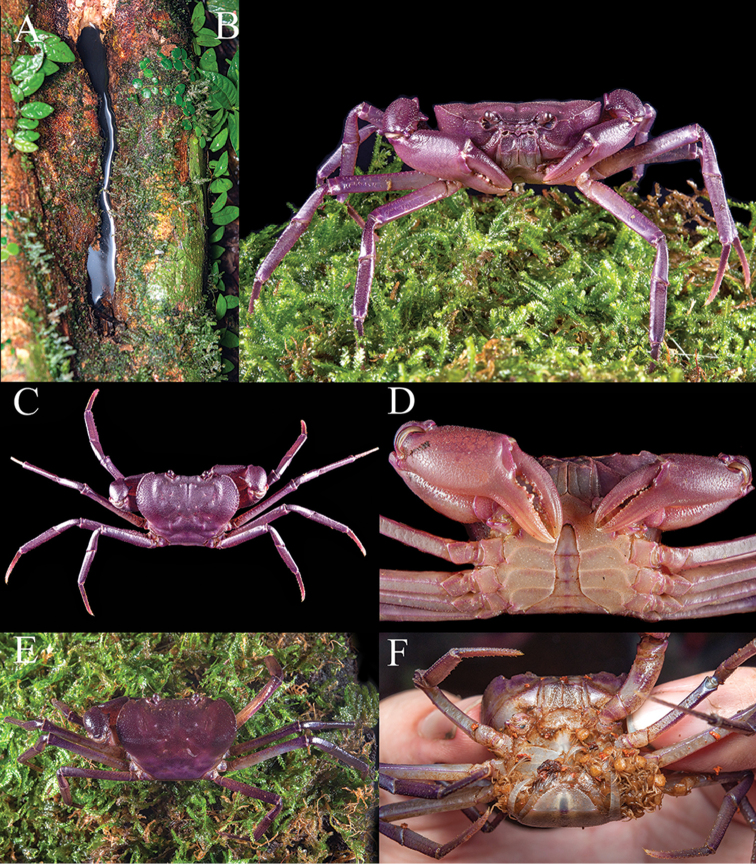
Habitat and life colour of *Arachnothelphusa terrapes*. **A** water-filled tree hole at base of tree in Danum Valley where crab was found **B** water filled tree hole where crabs were hiding **C, D** male (30.8 × 20.5 mm) (ZRC 2017.1205) **E, F** female (30.1 × 20.5 mm, with juvenile crabs) (ZRC 2017.1205).

The live coloration of this species observed in the recent pair of specimens is a uniform dark purple colour on the dorsal surface of the carapace, ambulatory legs and chelipeds, with a pale purple to dull white on the thoracic sternum, pleon and distal portions of the ambulatory legs and cheliped fingers (Fig. 5B–F). The dark purple colouration appears considerably darker when the animal is dry, explaining the original paler colour observation by Ng (1991).

####### Remarks.

Ng (1991) established *Arachnothelphsua* for several Bornean species previously classified as *Thelphusula* Bott, 1969, with Potamon (Potamon) melanippe De Man, 1899, as the type species. Currently, four other species are recognised: *A. kadamaiana* (Borradaile, 1900), *A. rhadamanthysi* (Ng & Goh, 1987), *A. terrapes* Ng, 1991, and *A. merarapensis* Grinang, Pui & Ng, 2015, all from northern Borneo. One species originally included by Ng (1991) in *Arachnothelphusa*, Parathelphusa (Liotelphusa) nobilii Colosi, 1920, was transferred to *Stygothelphusa* Ng, 1989, by Ng and Álvarez (2000) (see also Ng 2013; Ng and Grinang 2014).

*Arachnothelphusa terrapes* is easily distinguished from congeners by the deep U-shaped sinus separating the truncate external orbital tooth from the epibranchial tooth (Ng 1991: fig. 3). *Arachnothelphusa merarapensis* has a superficially similar anterolateral margin except that the two teeth are separated by an obtusely triangular broad cleft instead (Grinang et al. 2015: fig. 1A, B). Other congeners have the epibranchial tooth separated by a V-shaped notch or the margin is almost entire (De Man 1899: pl. 9; Ng and Goh 1987: pl. 3A; Ng 1991: fig. 1A; Grinang et al. 2015: fig. 6A).

The biology of species of *Arachnothelphsua* is not well known. All known species are represented by only very few specimens (Ng 1991) and there is often no accompanying ecological data. *Arachnothelphsua melanippe* and *A. kadaimana* were both described without any indication of their biology (De Man 1899; Borradaile 1900). Grinang et al. (2015) reported on a female specimen from Poring in Sabah but there was no information on where it was found. In the ZRC there are two lots of *A. kadaimana* (ZRC 2009.0094, ZRC 2002.0097) from Sabah, also without specific habitat data. A female specimen of *Arachnothelphusa*, close to but not conspecific with *A. kadaimana* (ZRC 2002.0098) from Bako National Park in Sarawak was collected from a tree trunk (I. Das, per. comm.). *Arachnothelphsua terrapes* was found low on shrubs (Ng 1991) while *A. rhadamanthysi* was collected on a stalagmite wall inside a cave (Ng and Goh 1987). The most detailed account so far was that by Grinang et al. (2015) for *A. merarapensis* from Merarap Hot Springs in Sarawak, who obtained the species from low tree holes approx. 150 cm from the ground. It is not known if the crabs live in phytotelms higher up on the forest trees. *Arachnothelphsua rhadamanthysi* has since been photographed by naturalists in the forested area outside Gomantong caves where it was first found, suggesting it is only a facultative cave dweller (Christensen 2015).

*Arachnothelphusa terrapes* was described from a pair of specimens, the first, a female collected in 1989 which moulted shortly after capture and died, leaving both the animal and exuvium in poor condition. The male holotype was collected a year later from a dry tree stump, with the live coloration being a deep reddish brown on dorsal surfaces, chelipeds and legs (Ng 1991: 11). In view of the present observations of this species as a tree hole specialist, it is likely the holotype male was only taking temporary refuge in the tree stump when it was found.

Two individuals of *A. terrapes* were observed at 0030 hours in Danum Valley, less than 50 m apart. The first, a large adult male was observed at the edge of a water-filled hole on a tree buttress, roughly 35 cm above the ground (Fig. 5A). A second, a female carrying newly hatched young under its pleon, was found inside a water filled tree hole approx. 150 cm above ground (Fig. 5F). This species is nocturnal and highly sensitive to light, swiftly retreating into their holes when disturbed. Additional observations by other naturalists who have photographed this species in Danum Valley suggest it is always found on trees and never on the forest floor itself (unpublished data). It is clear that *A. terrapes* is a true phytotelm species and predominantly arboreal in nature, living exclusively in tree-holes; although they will move in search of other tree holes if the one they are residing begins to dry up or when searching for a mate. All specimens have been observed on the lower parts of trees and it is not known if they climb much higher up. All specimens recorded so far have been solitary. The present observation of an adult female carrying young (Fig. 5F) is notable and confirms the species breeds in the phytotelm.

The biology of obligate arboreal crabs has been discussed at length by Sivasothi et al. (1993), Sivasothi (2000), Cumberlidge et al. (2005), Fratini et al. (2005), Grinang et al. (2015), Ng et al. (2015), Wehrtmann et al. (2016) and Kumar et al. (2017). While most are primary freshwater crabs (sensu Yeo et al. 2014) of the families Potamidae and Gecarcinucidae, members of two South East and East Asian sesarmid genera, *Geosesarma* De Man, 1892, and *Scandarma* Schubart, Liu & Cuesta, 2003, are primarily arboreal in habits (see Schubart et al. 2003; Naruse and Ng 2007; Ng 2017). There are of course some species of freshwater crabs that occasionally climb trees and use phytotelms but can also be found on the forest floor or nearby streams, and thus are not obligate arboreal species. In Asia, *Sundathelphusa celer* (Ng, 1991) (Gecarcinucidae), from the Philippines was collected in a tree hollow above ground, but it is not certain if it is a wholly arboreal species (Ng 2010). *Perbrinckia scansor* (Ng, 1995) from Sri Lanka has also been noted to have arboreal tendencies but is clearly a terrestrial species that occasionally climbs trees (Ng 1995: 183; Ng and Tay 2001: 148–149). In Hainan, China, some species of *Neotiwaripotamon* Dai & Naiyanetr, 1994, are known to be primarily arboreal (unpublished data; Shih 2008). In India, the only known true arboreal phytotelm species is the recently described *Kani maranjandu* Kumar, Raj & Ng, 2017.

#### Genus *Terrathelphusa* Ng, 1989

##### Type species.

*Geothelphusa kuhli* De Man, 1883, by original designation.

###### Terrathelphusa secula

Taxon classificationAnimaliaDecapodaGecarcinucidae

Ng & Tan, 2015

Figure 6E

Terrathelphusa secula Ng & Tan, 2015: 447, figs 1–3.

####### Material examined.

Holotype male (29.2 × 20.4 mm) (ZRC 2018.0297), found dead in pool adjacent to Borneo Rainforest Lodge, next to Danum Valley Conservation Area, Lahad Datu, Sabah, 4°58.2'N 117°41.4'E, ca. 600 m asl, East Malaysia, Borneo, coll. local ranger, 28 May 2015.

####### Colour.

The freshly dead type specimen was described as dark brown overall (Ng and Tan 2015: 448).

####### Remarks.

The species was described from just outside the Danum Valley Conservation Area by Ng and Tan (2015) from a recently dead specimen. *Terrathelphusa* species are difficult to collect due to their secretive terrestrial habits and tendency to dig deep burrows, coming out only at night and during the wet season (Grinang and Ng 2015).

*Terrathelphusa* was established by Ng (1989c) for a group of terrestrial species from Java and Borneo (type species *Geothelphusa kuhli* De Man, 1883, from Java) and now contains 11 species (Ng et al. 2008; Ng and Tan 2015); although the genus is probably not monophyletic (unpublished data). *Terrathelphusa secula* is unusual among congeners in possessing a very ovate carapace and a G1 that is elongate with a long and strongly curved terminal segment (Ng and Tan 2015).

#### Genus *Parathelphusa* H. Milne Edwards, 1853

##### Type species.

*Parathelphusa tridentata* H. Milne Edwards, 1853, by subsequent designation (Rathbun 1905).

###### Parathelphusa valida

Taxon classificationAnimaliaDecapodaGecarcinucidae

Ng & Goh, 1987

Figure 6C, D

Parathelphusa valida Ng & Goh, 1987: 317, pls 1, 2; fig. 1; Ng 1995: 79; Tan and Ng 1997: 555; Ng et al. 2008: 71; Klaus et al. 2013 68.

####### Material examined.

Holotype: male (40.0 × 30.0 mm) (ZRC 1989.2024), stream outside Simud Hitam Cave, Gomantong, Sabah, Borneo, ca 5°33'N 118°06'E, coll. P. Chapman, 27 March 1986. Paratypes: 1 male (ZRC 1989.2192), Simud Puteh Cave, Gomantong, Sabah, Borneo coll. P. Chapman, 27 March 1986; 2 males (ZRC 1990.0445–0446), 1 male (ZRC 1989.3402), Sungei Madai, in stream just outside Madai Caves, Kunak, Lahad Datu district, Sabah, Borneo, ca. 04°44'N 118°12'E, coll. January 1985; 1 male (73.0 × 73.0 mm) (ZRC 1990.0444), Sungei Binuang, stream adjacent to Baturung Caves, Kunak, Lahad Datu district, Sabah, Borneo, 4°43'N 117°59'E, coll. R. Goh, 20 March 1985. Others: 1 female (ZRC 1989.3403), Sungei Madai, in stream just outside Madai Caves, Kunak, Lahad Datu district, Sabah, Borneo, ca. 04°44'N 118°12'E, coll. R. Goh, January 1985; 1 male (ZRC 1996.1897), Gomantong Caves Sabah, coll. C.L. Chan, January 1995; 1 male, 1 female (ZRC 2009.0091), Gomantong Caves, coll. D. Chia, 21 December 1999; 1 female (ZRC 1996.1999), Sungei Binuang, Banturung Caves, Lahad Datu, coll. R. Goh, 20 March 1989; 1 male (ZRC 1990.0571), Danum Valley, Sabah, coll. R. Stuebing, 1980s; 1 male (ZRC 1990.0568), Danum Valley, Lahad Datu, Sabah, coll. R. Stuebing, 23 July 1989; 1 male, 2 females (ZRC 1008.1346), Sungei Palun Tambun, tributary of Sungei Segama, upstream of Danum Valley Field Centre, Lahad Datu, Sabah, coll. H.H. Tan et al., 1 October 1996; 2 males, 5 females (ZRC 1996.1998), Kallang Sebaru stream, 4°58'4.8"N, 107°48'56.5"E, Danum Valley, Lahad Datu, Sabah, coll. H.H. Tan et al., 1 October 1996; 1 male, 1 female (ZRC 1996.1997), Sepat Kalisun, Danum Valley, Lahad Datu, Sabah, coll. H.H. Tan et al., 1 October 1996; 2 males (ZRC 1996.1995) Ca Gin Stream Right, 4°59'8.5"N, 107°54'5.1"E, Danum Valley, Lahad Datu, Sabah, coll. H.H. Tan et al., 2 October 1996; 2 females (ZRC 1996.2000), Sungei Bole Ketabil tributary, 4°57'33.5"N, 117°51'34.1"E, Danum Valley Field Centre, Lahad Datu, Sabah, coll. H.H. Tan et al., 2 October 1996; 1 male, 2 females (ZRC 1996.1994), Sungei Bole Ketabil tributary, 4°57'33.5"N, 117°51'34.1"E, Danum Valley Field Centre, Lahad Datu, Sabah, coll. H.H. Tan et al., 2 October 1996; 2 males, 2 females (ZRC 2010.0045), West Six stream, tributary of Sungei Segama, 600 m inside conservation area, coll. H.H. Tan et al., 4 October 1996; 1 male, 1 female (ZRC 1996.2004), West Eight, forest stream, 800 m into conservation area, tributary of Sungei Segama, Danum Valley Field Centre, Lahad Datu, Sabah, coll. H.H. Tan et al., 4 October 1996; 2 males (ZRC 2009.0309), Sungei Palum Tambum, near Danum Valley Field Centre, Lahad Datu, Sabah, coll. K. Martin-Smith, 9 October 1996; 1 female (34.7 × 28.4 mm) (ZRC 2017.1268), Danum Valley Field Centre, in forest streams, Lahad Datu, Sabah, Borneo, at night, coll. locals, 20 July 2017; 1 male (27.8 × 22.7 mm), 1 young female (17.4 × 5.3 mm) (ZRC 2017.1269), Danum Valley Field Centre, just outside dorms in streams, at night, Lahad Datu, Sabah, Borneo, coll. 22 July 2017; 1 male, 1 female (ZRC 2008.483), Maliau Basin, stream draining into Sungei Maliau, coll. S.H. Tan and T.H.T. Tan, 15–17 May 1996; 2 males, 1 female (ZRC 2008.0607), Maliau Basin, tributary of Sungei Maliau, adjacent Camp 88, coll. S.H. Tan and T.H.T. Tan 15–17 May 1996; 1 male, 1 female (ZRC 1997.0104), Maliau Basin, coll. S.H. Tan and T.H.T. Tan, 13–17 May 1996; 1 female (ZRC 1989.2194), Maliau Basin, Sabah, coll. Sabah Foundation Expedition 1988; 1 male, 2 females (ZRC 1996.2005), Tawau Plateau, Telupid Sandakan stream, coll. R. Goh, 1990; 5 males (ZRC 2008.1345), stream by Air Panas, near base of Tawau Hills Park, coll. H.H. Tan et al., 5 October 1996; 1 male 4 females (ZRC 1996.2008), Tawau, Sabah, 4°18'03"N, 117°54'20.7"E, coll. H.H. Tan et al., 5 October 1996; 2 males, 4 females (ZRC 1996.2001), Tawau, Sungei Matarid, Gua Madai, Jalan Madai, 4°43'8.7"N, 118°9'14.7"E, Tawau, Sabah, H.H. Tan et al., 6 October 1996; 1 male (ZRC 1994.4201), Danau Biandum, Kinabatangan River, Sabah, coll. S.H. Tan et al., 8 April 1994.

####### Colour.

**Figure 6.**
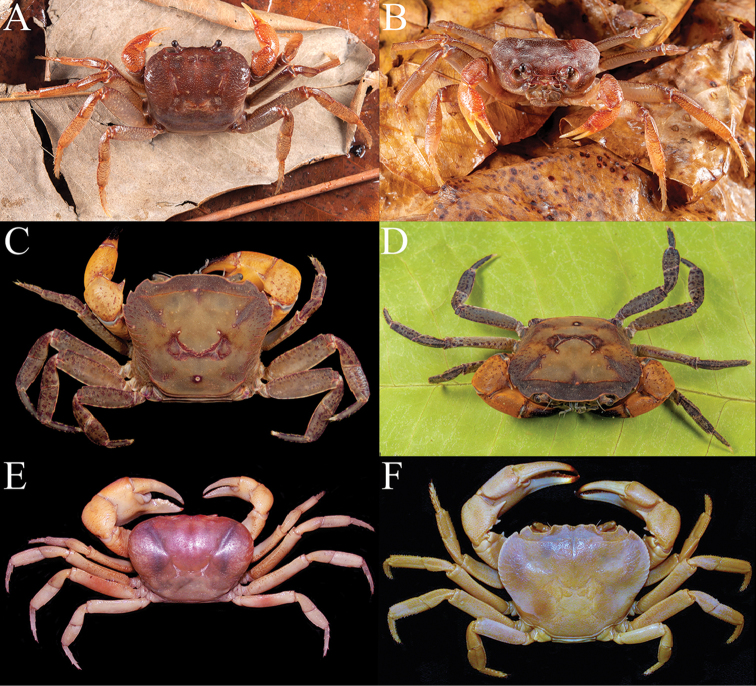
**A, B** *Thelphusula dicerophilus*, female (26.1 × 21.3 mm) (ZRC 2017.1047) (in situ) **C, D** *Parathelphusa valida*, male (27.8 × 22.7 mm) (ZRC 2017.1269) **E** *Terrathelphusa secula*, holotype male (29.2 × 20.4 mm) (ZRC 2018.0297) (preserved colour) **F** *Isolapotamon ingeri*, male (57.4 × 43.5 mm) (ZRC 1997.0799) (preserved colour).

Fresh specimens have an olive-brown carapace with the grooves and striae reddish brown; the ambulatory legs are brown with specks of reddish brown; and the chelipeds are orange, with the fingers black except for the orange tip (Fig. 6C, D).

####### Remarks.

The recently collected specimens agree well with the published descriptions and figures of the species, originally described from Gomantong, Bettontan and Lahad Datu in Sabah (Ng and Goh 1987). The species has a wide range in eastern Sabah (see also Ng 1995; Tan and Ng 1997).

*Parathelphusa valida* occurred syntopically with *T. capillodigitus* sp. n. and was present in a variety of habitats including jungle streams, swampy areas and on the forest floor. It has not been previously formally recorded from Danum Valley, which is surprising, considering it is by far the most common species there and there are many specimens in the museum dating back to the 1980s.

### Family Potamidae Ortmann, 1896

#### Genus *Isolapotamon* Bott, 1968

##### Type species.

*Potamon anomalus* Chace, 1938, by original designation.

###### Isolapotamon ingeri

Taxon classificationAnimaliaDecapodaGecarcinucidae

Ng & Tan, 1998

Figure 6F

Isolapotamon sp. – Ng and Goh 1987: 328, pl. 3, D.
Isolapotamon ingeri Ng & Tan, 1998: 66, figs 6E–H, 7; Ng et al. 2008: 163.

####### Material examined.

Holotype: male (44.3 × 33.3 mm) (ZRC 1997.0796), Sungei Tawau, Tawau Hills Park, Tawau, Sabah, coll. P. Yam, 14 December 1991. Paratypes: 1 female (41.4 × 31.0 mm) (ZRC 1997.0797), same data as holotype. Others: 1 male, 2 females, 1 juvenile (ZRC 2000.2217), Lower Segama River, Danum Valley Field Centre, Lahad Datu, Sabah, coll. K. Martin-Smith, June 1996; 1 male (57.4 × 44.8 mm) (ZRC 1997.0798), Sungei Palum Tambum, near Danum Valley Field Centre, Lahad Datu, Sabah, coll. K. Martin-Smith, August 1996; 5 males (largest 57.4 × 43.5 mm), 1 female (ZRC 1997.0799), Sungei Palum Tambun, near Danum Valley Field Centre, Lahad Datu, Sabah, coll. K.M. Martin-Smith, 9 October 1996; 1 female (ZRC 2000.2218), Sepat Kalisun, stream 200 m from 4°58'04.8"N, 117°48'56.5"E, Danum Valley Field Centre, Lahad Datu, Sabah, coll. H.H. Tan et al., 1 October 1996; 1 male, 2 females (ZRC 2000.2210), Sungei Bole Ketabil tributary, 4°57'33.5"N, 117°51'34.1"E, Danum Valley Field Centre, Lahad Datu, Sabah, coll. H.H. Tan et al., 2 October 1996; 1 male, 2 females (ZRC 2000.2220), Cabin Stream, 50 km on road to Danum Valley Conservation Area, drains from Bukit Rafflesia, Lahad Datu, Sabah, coll. H.H. Tan et al., 2 October 1996; 1 male (ZRC 2000.2221), West Eight, forest stream, 800 m into conservation area, tributary of Sungei Segama, Danum Valley Field Centre, Lahad Datu, Sabah, coll. H.H. Tan et al., 4 October 1996; 2 females (ZRC 2008.0431), Danum Valley Rainforest Lodge, 5°03'2.9"N, 117°34'34.8"E, Lahad Datu, Sabah, 3 October 1996; 1 female (56.0 × 42.0 mm) (ZRC 1989.3419), Sungei Madai, Madai Caves, Sabah, coll. R. Goh, 27 January 1985; 1 male (ZRC 1997.0802), Tawau, Sungei Matarid, Gua Madai, Jalan Madai, 4°43'8.7"N, 118°9'14.7"E, Tawau, Sabah, H.H. Tan et al., 6 October 1996.

####### Colour.

The colour in life is dark green overall (H.H. Tan, pers. comm.).

####### Remarks.

*Isolapotamon ingeri* belongs to the same group of species as *I. kinabauense* (Rathbun, 1904) and *I. anomalum* (Chace, 1938) (both from the Mount Kinabalu area in northern Sabah), with the distal part of the terminal segment of the G1 expanded and flap-like (Ng and Tan 1998). Like these species, *I. ingeri* usually occurs in large streams and rivers with large rocks and fast flowing water.

### Family Sesarmidae Dana, 1851

#### Genus *Geosesarma* De Man, 1892

##### Type species.

Sesarma (Geosesarma) nodulifera De Man, 1892, by subsequent designation (Serène and Soh 1970).

###### Geosesarma danumense

Taxon classificationAnimaliaDecapodaGecarcinucidae

Ng, 2002

Figure 7A–C

Geosesarma danumense Ng, 2002: 303, figs 1–3; Ng et al. 2008: 220.

####### Material examined.

Holotype: male (14.8 × 14.6 mm) (ZRC 2017.1298), in pitfall trap, primary forest, Danum Valley Field Centre, Sabah, Malaysia, coll. C. Colón, 22 November 1996. Others: 1 ovigerous female (14.9 × 15. mm) (ZRC 2017.1273), in water filled rotting log, Nature Trail, Danum Valley Field Centre, Sabah, Borneo, coll. local rangers, 21 July 2017.

####### Comparative material.

*Geosesarma sabanum* Ng, 1992: holotype male (13.1 × 13.6 mm) (ZRC 2018.0296), on leaf of herb in forest, ca. 50 m from nearest stream, Tawau Hills Park, eastern Sabah, Malaysia, Borneo, coll. R.F. Inger, 3 November 1991.

####### Colour.

**Figure 7.**
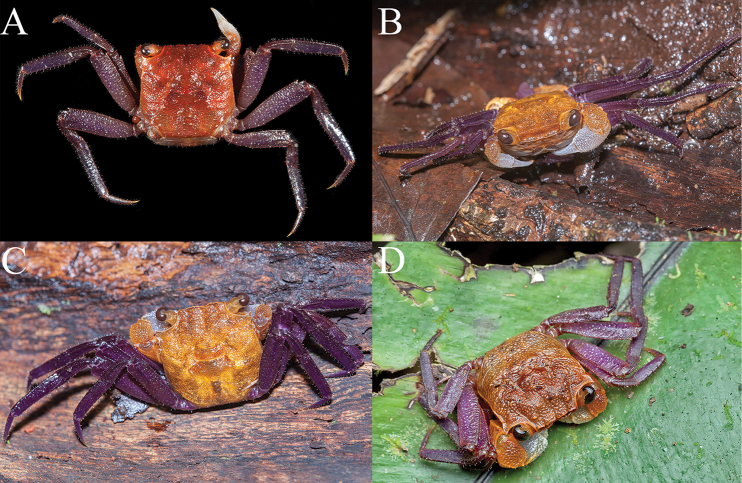
**A** *Geosesarma danumense*, ovigerous female (14.9 × 15.1 mm) (ZRC 2017.1273) **B, C** *G. danumense*, male (carapace width ca. 1.5–2.0 cm), on log approx. 30 cm from ground, ca. 30 m from river flowing through Danum Valley Field Centre, 10 pm, 25 July 2013, specimen not collected (photographs: Marcus Ng) **D** *G. sabanum*, male, on leaf above ground, Tawau Hills National Park, specimen not collected (photograph Ying Seawei).

*Geosesarma danumense* has a dark yellow to orange carapace, purple ambulatory legs with scattered white specks, orange merus and carpus of the chelipeds, with the palm and fingers white (Fig. 7A–C). The eggs of the recently collected ovigerous female were observed to be a bright reddish orange in colour and large in size, indicating that the development is probably direct (see Soh 1969; Ng and Tan 1995). The live coloration is very similar to the related *G. sabanum* Ng, 1992 from Tawau (Fig. 7D).

####### Remarks.

*Geosesarma danumense* and *G. sabanum* are morphologically close, although the external orbital tooth of the latter species is proportionately more slender, the frontal margin less truncate, the ambulatory meri proportionately shorter, and most significantly the corneous distal part of the G1 is proportionately longer (see Ng 2002).

The holotype of *G. danumense* was obtained from a pitfall trap while the recent large ovigerous female (ZRC 2017.1273) was collected from under a rotting log. Specimens have also been observed climbing small shrubs. In this respect, it probably has similar habits to *G. sabanum* from Tawau which has been observed by the second author to hide between the leaves of *Pandanus* sp. during the day, emerging only at night to forage on low lying vegetation and occasionally amongst leaf litter (unpublished data). The terrestrial habits of *G. danumense* and *G. sabanum* probably parallel those known for species in Peninsular Malaysia and Indonesian Kalimantan (Ng 2015, 2017).

Manuel-Santos et al. (2016: 336) commented that the three species of *Geosesarma* on Palawan Island in the Philippines, *G. lawrencei* Manuel-Santos & Yeo, 2007, *G. batak* Manuel-Santos, Ng & Freitag, 2016, and *G. tagbanuana* Manuel-Santos, Ng & Freitag, 2016, are morphologically very similar to the Sabahan *G. danumense* and *G. sabanum*, notably in their relative large adult size and long slender ambulatory legs. Their G1 structures, however, are very different, with those of the latter two species proportionately much shorter and stouter (cf. Ng 1992, 2002; Manuel-Santos and Yeo 2007; Manuel-Santos et al. 2016).

### Key to freshwater crabs in the Danum Valley Conservation Area

**Table:** 

1	Third maxillipeds forming median rhomboidal gap when fully closed; carapace frontal margin with 4 distinct truncate lobes; cornea large, appearing bulbous in life; frontal and lateral surfaces of carapace with net-like pattern of short setae; exopod of third maxilliped without flagellum; terrestrial species	***Geosesarma danumense***
–	Third maxillipeds closing without any median rhomboidal gap; carapace frontal margin entire with 2 weakly separated rounded lobes; eyes not swollen in life; frontal and lateral surfaces of carapace may be granular but never with net-like pattern of short setae; exopod of third maxilliped with distinct flagellum; terrestrial and aquatic species	**2**
2	Anterolateral margin of carapace with 3 well-defined, sharp teeth (including external orbital tooth)	***Parathelphusa valida***
–	Anterolateral margin of carapace rounded or straight, entire or with at most one tooth (external orbital tooth)	**2**
3	Frontal margin and orbits of carapace appears sunken in from dorsal view; ambulatory legs very elongate, longest leg 4–5 times longer than carapace length; lives in tree-holes	***Arachnothelphusa terrapes***
–	Frontal margin and orbits of carapace level with sides from dorsal view; ambulatory legs proportionately much shorter; free-living	**4**
4	Anterolateral carapace margin with a distinct epibranchial tooth clearly separated from external orbital tooth by V-shaped notch; G1 with neck-like median section and rectangular flap distally; lives under rocks in fast flowing water	***Isolapotamon ingeri***
–	Anterolateral carapace margin appears entire or with a low epibranchial tooth barely separated from external orbital tooth; G1 gradually tapering towards tip, straight or curved; terrestrial, semiterrestrial, in swampy areas or slow flowing forest streams with leaves and detritus	**5**
5	Carapace relatively flat, not prominently raised; gastric regions with distinct transverse striae; fingers of adult male chelipeds with dense mat of short setae; mostly aquatic species	***Thelphusula capillodigitus***
–	Carapace prominently raised, appears swollen; gastric regions appears smooth, without prominent transverse striae; fingers of adult male chelipeds granulated, without setae; terrestrial to semiterrestrial species	**6**
6	Carapace almost squarish to slightly rectangular; G1 terminal segment elongate, straight	***Thelphusula dicerophilus***
–	Carapace transversely ovate, egg-shaped; G1 terminal segment strongly curved, hook-like	***Terrathelphusa secula***

## Supplementary Material

XML Treatment for Thelphusula capillodigitus

XML Treatment for Thelphusula dicerophilus

XML Treatment for Arachnothelphusa terrapes

XML Treatment for Terrathelphusa secula

XML Treatment for Parathelphusa valida

XML Treatment for Isolapotamon ingeri

XML Treatment for Geosesarma danumense
